# Epoxidation and etherification of alkaline lignin to prepare water-soluble derivatives and its performance in improvement of enzymatic hydrolysis efficiency

**DOI:** 10.1186/s13068-016-0499-9

**Published:** 2016-04-14

**Authors:** Changzhou Chen, Mingqiang Zhu, Mingfei Li, Yongming Fan, Run-Cang Sun

**Affiliations:** Beijing Key Laboratory of Lignocellulosic Chemistry, Beijing Forestry University, Beijing, 100083 China; College of Forestry, Northwest A&F University, Yangling, 712100 China

**Keywords:** Lignin, Epoxidation, Etherification, Emulsification, Surface tension, Enzymatic hydrolysis, Ethanol fermentation

## Abstract

**Background:**

Due to the depletion of fossil resources and their environmental impact, woody biomass has received much attention as an alternative resource. Lignin, as the third most abundant biopolymer from biomass, is now considered as an excellent alternative feedstock for chemicals and materials. The conversion of lignin to the value-added products is a key process to achieve an integrated biorefinery of woody biomass. Among these value-added products, lignin-based derivatives with good surface activity can be applied to enhance the conversion of cellulose into fermentable sugars, which not only decrease the cost of bioethanol production, but also reduce the environmental pollution and green house effect resulting from the burning of fossil resources.

**Results:**

Water-soluble alkaline lignin was synthesized by the reaction between polyethylene glycols (PEG600 and PEG1000) and epoxy lignin. FT-IR and NMR analyses indicated that PEGs were successively introduced into epoxy alkaline lignin using potassium persulfate as a catalyst. Emulsification and surface activity tests indicated that the surface tension of the prepared lignin derivative solution was 43.30 mN/m at the critical micelle concentration (1.03 %). A stable emulsions layer was formed with hexanes and the emulsion particle diameter in the emulsion phase for all products was observed at 10–50 μm. The results of enzymatic hydrolysis indicated that the products derived from PEG1000-grafted lignin resulted in the highest increasing rate of 18.6 % of glucose yield during the enzymatic hydrolysis of hardwood bleached pulp. The results of fermentation experiments suggested that the product had no toxicity for fermentation micro-organisms.

**Conclusion:**

Water-soluble alkaline lignin derivatives were prepared through epoxidation and etherification, which are promising feedstocks for detergents, emulsifier, and additive to enhance enzymatic hydrolysis efficiency and ethanol fermentation.

**Electronic supplementary material:**

The online version of this article (doi:10.1186/s13068-016-0499-9) contains supplementary material, which is available to authorized users.

## Background

Currently, most of the carbon-based compounds manufactured in chemical industry are derived from petroleum. The possible routes to prepare chemicals, fuels, and solvents from biomass have received increased attention due to the rising cost and dwindling supply of oil [1–3]. Lignin, the third abundant carbon-rich substance from biomass, accounts for 20–30 % of wood [4]. It is a disordered polymer with structural diversity and heterogeneity, which consists of *p*-hydroxyphenyl (H), guaiacyl (G), and syringyl (S) units linked by various inter-unit linkages (e.g., β-O-4′, β-5′, β-β′, 5-5′, 5-O-4′, and β-1′.) [5, 6]. In addition, lignin serves as a glue embedding the polysaccharide network in the cell wall, which gives the cell wall rigidity, impermeability, and structural reinforcement [7, 8]. As a polyfunctional biomass monomer, lignin contains plentiful hydroxyl, carbonyl, carboxyl, and methyl groups. It is an exciting candidate for chemical reactions to produce new materials and chemicals [9] due to its active functional sites.

Low-molar mass phenolic compounds like vanillin, simple hydroxylated aromatics, and many other compounds can be obtained from depolymerization of lignin via pyrolysis, catalyzed depolymerization, gasification, oxidation, and liquefaction [10–12]. However, depolymerization of lignin is a negative energy process when comparing with lignin biosynthesis in nature. Therefore, a number of modification methods have been proposed to transform it into various lignin-based products. For example, the utilization of lignin to replace phenol for preparing lignin–phenol–formaldehyde resins has been investigated in our previous studies [13]. Lignin also acts as a polyol precursor in the synthesis of polyurethane (PU) involving the formation of urethane links via the reaction of OH groups with RN=C=O groups [14–16].

As a polyol rich in hydroxyl groups, lignin can react with other appropriate chemical compounds to introduce new chemical reactive sites (e.g., carboxylic, amine, and epoxy groups). Lignin-based epoxy resin monomer (LERM) can be prepared by the reaction of phenolic and alcoholic hydroxyl with epichlorohydrin. The lignin-based epoxy resin was produced through the crosslinking among the epoxy groups under the catalysis of curing agent [17]. Before the curing reaction, LERM with abundant epoxy groups gives a new kind of chemical reaction sites of lignin structure. Epoxy groups have a good reactivity with some compounds containing hydroxyl or amino groups [18, 19]. The introduction of epoxy groups into lignin gives certain lipophilic capacity to the lignin and makes it easily dissolvable in some common organic solvents (tetrahydrofuran, methylene chloride, acetone, chloroform, etc.) [17, 20]. It offers the possibility of more routes for the conversion of lignin into value-added products via chemical modification. On this basis, some of water-soluble functional groups (carboxyl, sulfonic, amino, and polyether ones) can be introduced into LERM to prepare certain water-soluble lignin derivatives (e.g., surfactants) with good surface activity.

With respect to the reactions of the phenolic hydroxyl or the ortho and para reactive place of phenolic hydroxyl, three types (cationic, anionic, and non-ionic forms) of water-soluble lignin derivatives with good surface activity have been investigated. Many studies have been conducted in the exploitation of anionic lignin derivatives in the past decades, but less in the two other types. The preparation routes of these types of water-soluble lignin derivatives are summarized below. One method for transforming lignin to a good water-soluble anionic surfactant is sulfonation and has been widely studied [21, 22], and the direct value of this kind of lignin-based surfactant has been realized on the market. In addition to the traditional applications (e.g., oil recovery sacrifice agent, pesticide, and dye dispersant), anionic lignin surfactant has been applied to the investigation of preparing high-quality graphene [23–25]. Cationic lignin-based surfactants with amino groups are synthesized mainly based on Mannich reaction with amine and formaldehyde [26]. Except for surfactants, the potential value-added applications of the cationic lignin-based surfactants can be used as polycationic materials and slow-release fertilizers [27]. Recently, water-insoluble lignin has been converted into non-ionic and amphiphilic lignin derivatives. This was achieved by introducing polyethylene glycol (PEGs) as a hydrophilic moiety into lignin through the reactions between lignin phenol hydroxyl and PEG with diglycidyl (PEGDE) and monoglycidyl (EPEG) groups or PEG-chlorohydrin [28, 29]. In addition to the conventional application, this kind of non-ionic lignin derivatives as a direct additive has a good contribution to the improvement of enzymatic hydrolysis and bioethanol production as well as the commercial non-ionic surfactants [24, 30, 31]. It can provide a great prospect of the cost reduction for bioethanol production.

In the present study, a new method was applied to prepare water-soluble lignin derivatives. LERM was prepared using an industrial alkaline lignin. Then, water-soluble lignin derivatives were synthesized by the reaction of the LERM with PEGs. The structures of the lignin samples were characterized by FT-IR and NMR, and the emulsifiability, surface activity as well as the effect on enhancing enzymatic hydrolysis and ethanol fermentation were investigated. The current study is meaningful for the high-value utilization of lignin.

## Results and discussion

### Preparation of LPEGs

**Fig. 1:**
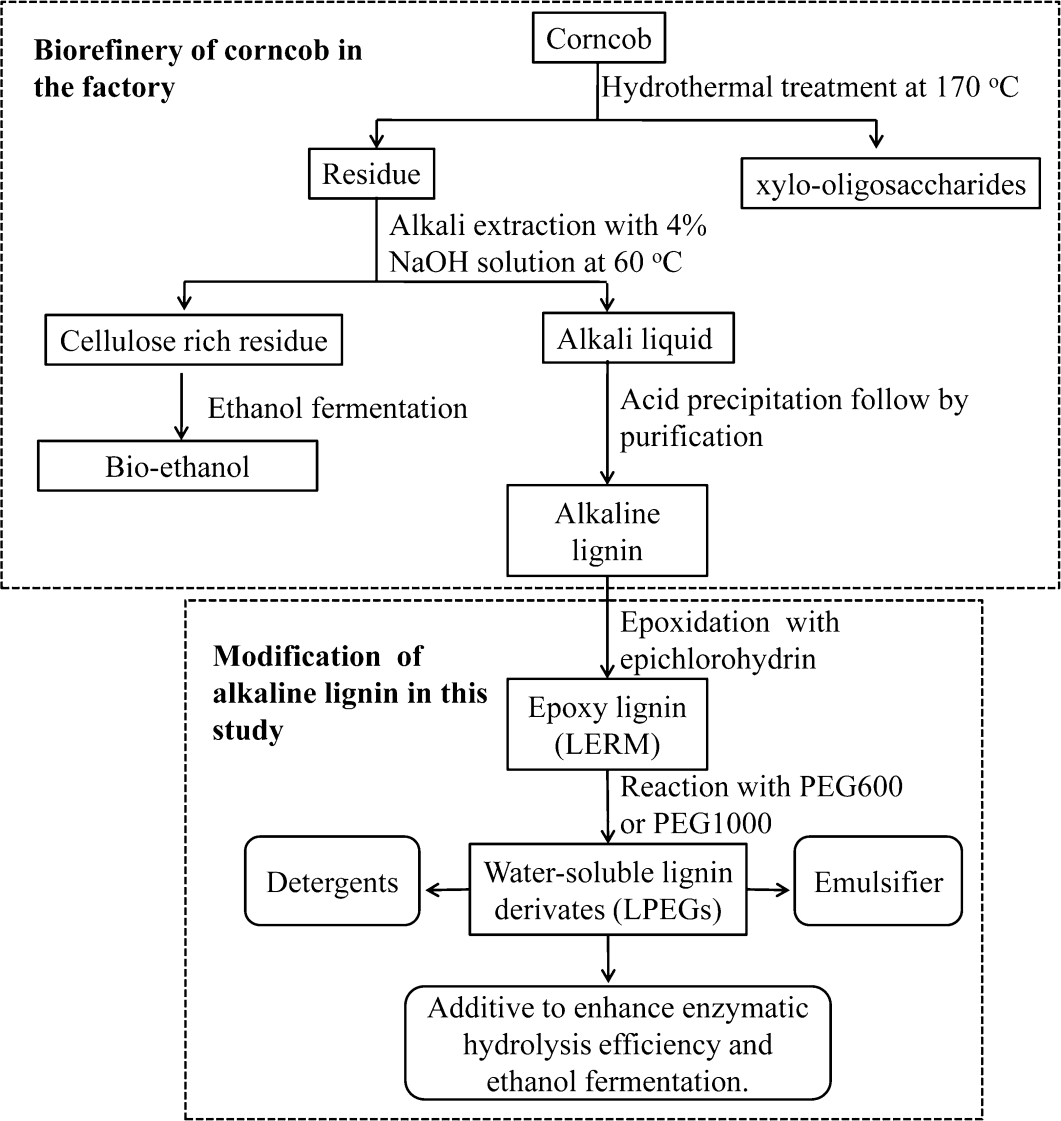
Overall process diagram including the general production of alkaline lignin from corncob in factory and the modification of the lignin in this study

The general production process of alkaline lignin is displayed in Fig. 1. First, the hemicelluloses of the corncob were degraded by hydrothermal treatment at 170 °C to obtain xylo-oligosaccharides. Second, the residual lignin was extracted with 4 % alkali solution at 60 °C and a cellulose-rich residue was obtained. And then, the final solid residue was used as a material for bioethanol production. The liquid, obtained from alkali treatment, was adjusted to acidic condition to precipitate the lignin. The alkaline lignin has a better compatibility with epichlorohydrin as compared to lignosulfonate. As seen in Additional file 1: Figure S1, it was difficult for lignosulfonates to be dissolved in epichlorohydrin before and after adding EDTA, which resulted in the inadequate chemical reaction between lignosulfonates and epichlorohydrin. Hence, to ensure the sufficient reaction of lignin with epichlorohydrin, the alkaline lignin was chosen as the raw material for chemical modification in the present work.

**Scheme 1:**
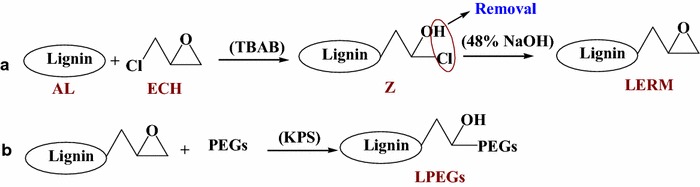
Synthesis route of LPEGs. **a** The epoxidation reaction of alkaline lignin. **b** Introduction of PEGs into lignin through the reaction of epoxy lignin with PEGs (*ECH* epichlorohydrin, *TBAB* tetrabutyl ammonium bromide, *Z* the intermediate of the ring-opened reaction of epichlorohydrin with lignin, *LERM* lignin-based epoxy resin monomer, *PEGs* PEG600 or PEG1000, *KPS* potassium persulfate, *LPEGs* water-soluble lignin derivatives)

The two general synthetic steps of LPEGs are shown in Fig. 1. First, the alkaline lignin was epoxidized with epichlorohydrin, and then the epoxy lignin was etherified with PEGs to obtain the water-soluble lignin derivatives (LPEGs). The solubility of LERM in organic solvent was associated with the content of epoxy groups. Because of the complex intrinsic structure of lignin, the epoxy group content in LERM derived from different kinds of lignin varied even at the same epoxidation condition. Scheme 1a shows the synthesis approach of LERM. As can be seen, the intermediate Z was formed by the reaction of epichlorohydrin with lignin along with ring-opening reaction under the catalysis of TBAB. The LERM was generated through the ring-closing reaction of Z by the removal of HCl under the catalysis of 48 % NaOH solution. In this study, to achieve sufficient reaction of LERM with PEGs, the epoxidation conditions of the raw material were investigated until a certain amount of LERM was completely dissolved in dichloromethane. For the LERM with good solubility in the organic solvent, petroleum ether was used to precipitate the LERM presenting in powder with yellow-brown color. The yield of LERM was 14.3 g obtained from the epoxidation reaction of 10.0 g of alkaline lignin. The epoxy value of LERM was 0.32 mol epoxy/100 g LERM, determined according to the method described by a previous report [17]. The molecular weight of LERM was 3000 g/mol, determined based on the GPC method. Self-polymerization of LERM and the grafting reaction of LERM with PEGs occurred simultaneously in the reaction system. When the common organic solvents (DMSO and DMF) were used as the solvent in the reaction between LERM and PEGs, a poor water-soluble and dichloromethane-soluble product was obtained, which suggested that the degree of polymerization among LERM units was larger than that of the reaction between LERM and PEGs. Thus, for the preparation of LPEGs, excessive amounts of PEGs (as solvent and reagent) were applied to react with LERM. Potassium persulfate, an initiator for monomer polymerization with oxidization-generated anionic free radicals (S_2_O_8_^2−^, 2·SO_4_^−^) under heating [32], was applied to catalyze the reaction of LERM with PEGs (see Scheme 1b). Furthermore, a part of the final products were not dissolved completely in the deionized water when the reaction was run at 100–120 °C. Therefore, relatively high temperatures were employed for the reactions (130–140 °C). The reaction condition, mass-average (Mw) and number-average (Mn) molecular weights, and the PEG content of the products are listed in Table 1. As can be seen, the Mw of all LPEG samples was lower than that of LERM (3000 g/mol) and close to the Mw of AL (850 g/mol) [6], which indicated that certain oxidation depolymerization reaction simultaneously occurred during the preparation of LPEGs resulting from the catalysis of KPS. The PEG content of LP600s (LP600_0.8_, LP600_1.0_, and LP600_1.2_) increased with the increase of the dosage of KPS. However, an increase in the amount of catalyst also strengthened the oxidation depolymerization of the products, which caused the decrease of the molecular weight of LP600_1.2_ as compared to LP600_1.0_. The increase of the reaction temperature also heightened the oxidation depolymerization of the products, resulting in a relatively smaller molecular weight of LP1000_140_ (890 g/mol) as compared to LP1000_130_ (1290 g/mol). The yields of LP600_0.8_, LP600_1.0_, LP600_1.2_, LP1000_130_, and LP1000_140_ were 3.7, 4.1, 4.3, 4.2, and 4.5 g, respectively, obtained from the reaction of 2.0 g of LERM with PEG600 or PEG1000.

**Table 1:** Reaction conditions, mass-average (Mw) and number-average (Mn) molecular weights of products, and the content of PEG

Sample^a^	Reaction conditions		Molecular weight^b^			Content of PEG (%)
	Temperature (°C)	Catalyst (g)	Mn (g/mol)	Mw (g/mol)	Mw/Mn	
LP600_0.8_	130	0.8	770 ± 7	900 ± 7	1.16	63.0
LP600_1.0_	130	1.0	800 ± 14	930 ± 20	1.15	65.0
LP600_1.2_	130	1.2	780 ± 14	900 ± 7	1.14	67.0
LP1000_130_	130	1.2	890 ± 14	1290 ± 28	1.43	68.0
LP1000_140_	140	1.2	770 ± 7	890 ± 14	1.16	70.0

^a^LP600_0.8_, LP600_1.0_, and LP600_1.2_ represent the products obtained from the reaction between LERM and PEG600 at 130 °C using 0.8, 1.0, and 1.2 g catalyst, respectively; LP1000_130_ and LP1000_140_ represent the products obtained from the reaction between LERM and PEG1000 at 130 and 140 °C using 1.2 g catalyst, respectively

^b^Data represented are mean values and standard deviations of the results obtained from the duplicated determination

### FT-IR spectra analysis

**Fig. 2:**
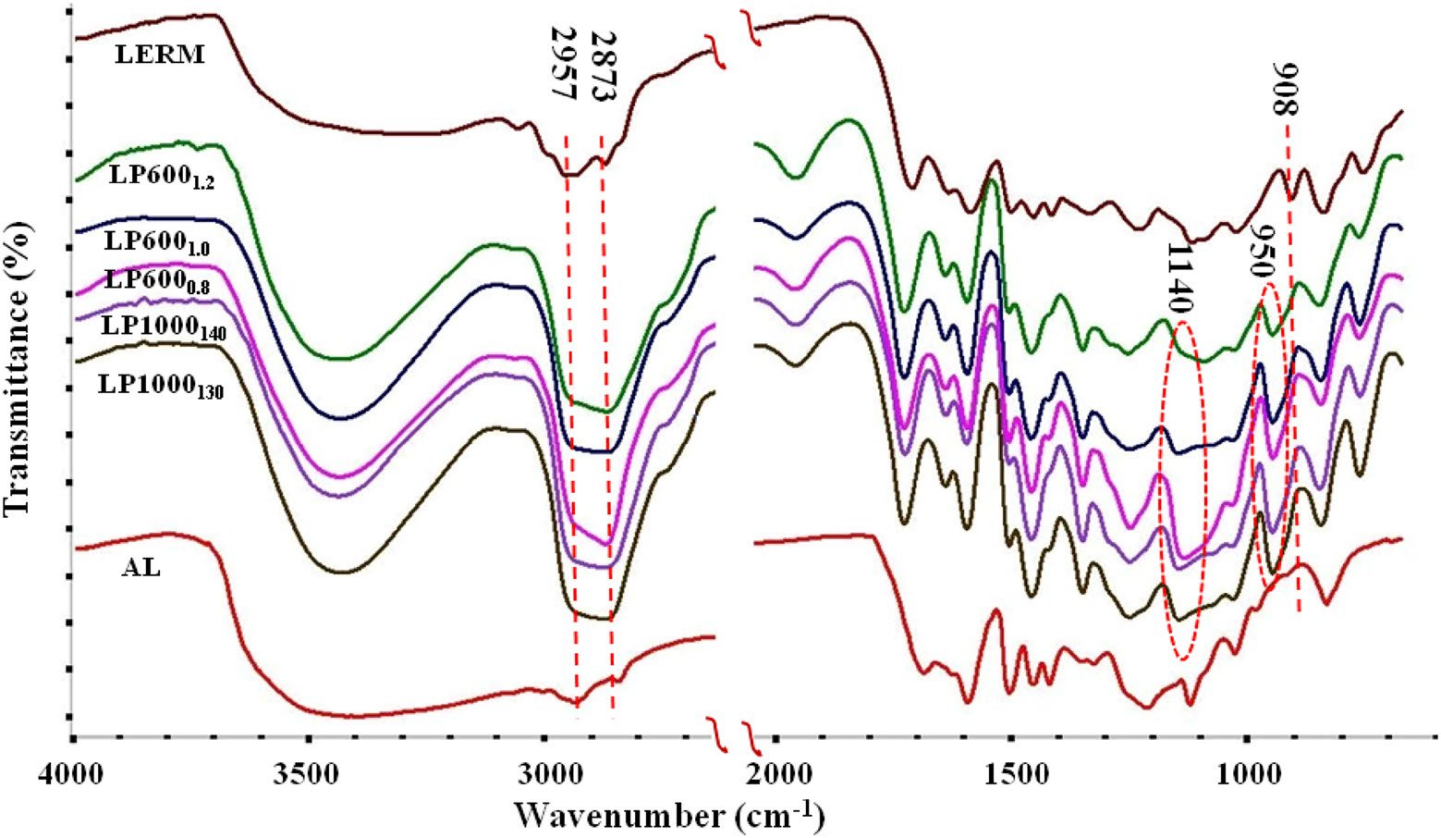
FT-IR spectra of alkaline lignin, LERM, and LPEGs

As shown in Fig. 2, the characteristic absorptions of lignin were located at 1600, 1510, and 1420 cm^−1^ in all samples. For the LERM, the absorptions of the specific epoxy groups appeared in the spectrum. As compared to AL, a new peak at 908 cm^−1^ corresponding to the asymmetric vibration of the epoxy groups appeared in LERM [17]. The lower OH stretching band intensity of LERM compared with AL was ascribed to the reaction of lignin OH with epichlorohydrin. During the synthesis of LPEGs, tertiary hydroxyl groups were derived from the ring-opening reaction of the epoxy groups in the LERM. Certain carboxy groups were also generated from the oxidation of products by the catalysis of KPS. Thus, the intensity of the bands attributing to OH in LPEGs was stronger than that of AL and LERM. As compared to AL and LERM, the successful introduction of PEGs into lignin was justified by the stronger bands at 1140 and 950 cm^−1^ (C–O–C stretching vibration) and the increased intensity of the bands at 2925 and 2873 cm^−1^ (C–H stretching of methyl and methylene) in LPEGs. Additionally, the absorption peak at 908 cm^−1^ appeared in the LERM but disappeared in LPEGs, which further illustrated the successful epoxidation of alkaline lignin and the reaction between PEGs and LERM.

### ^1^H-NMR and ^13^C-NMR spectra analysis

**Fig. 3:**
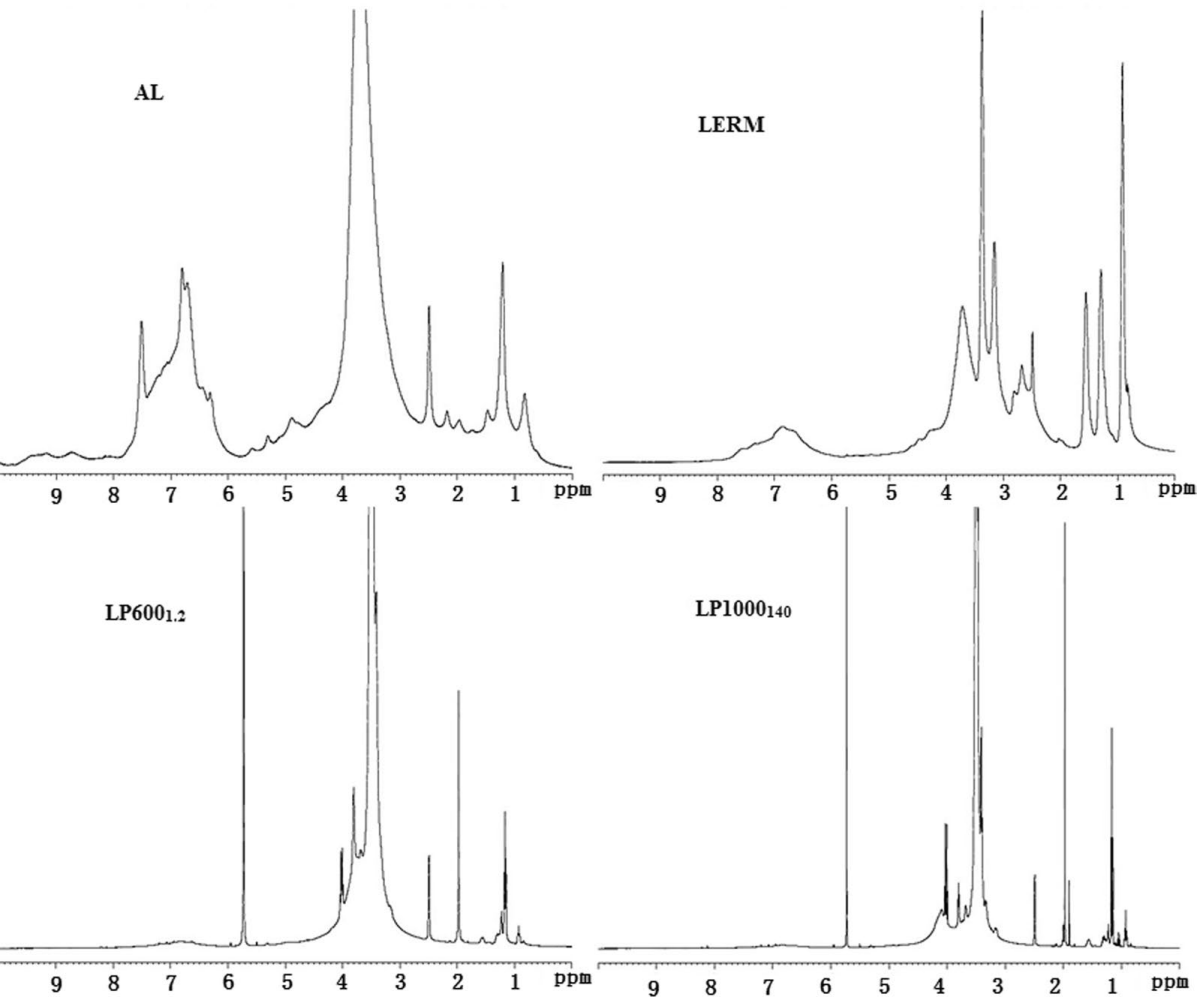
^1^H-NMR spectra of AL, LERM, LP600_1.2_, and LP1000_140_

**Fig. 4:**
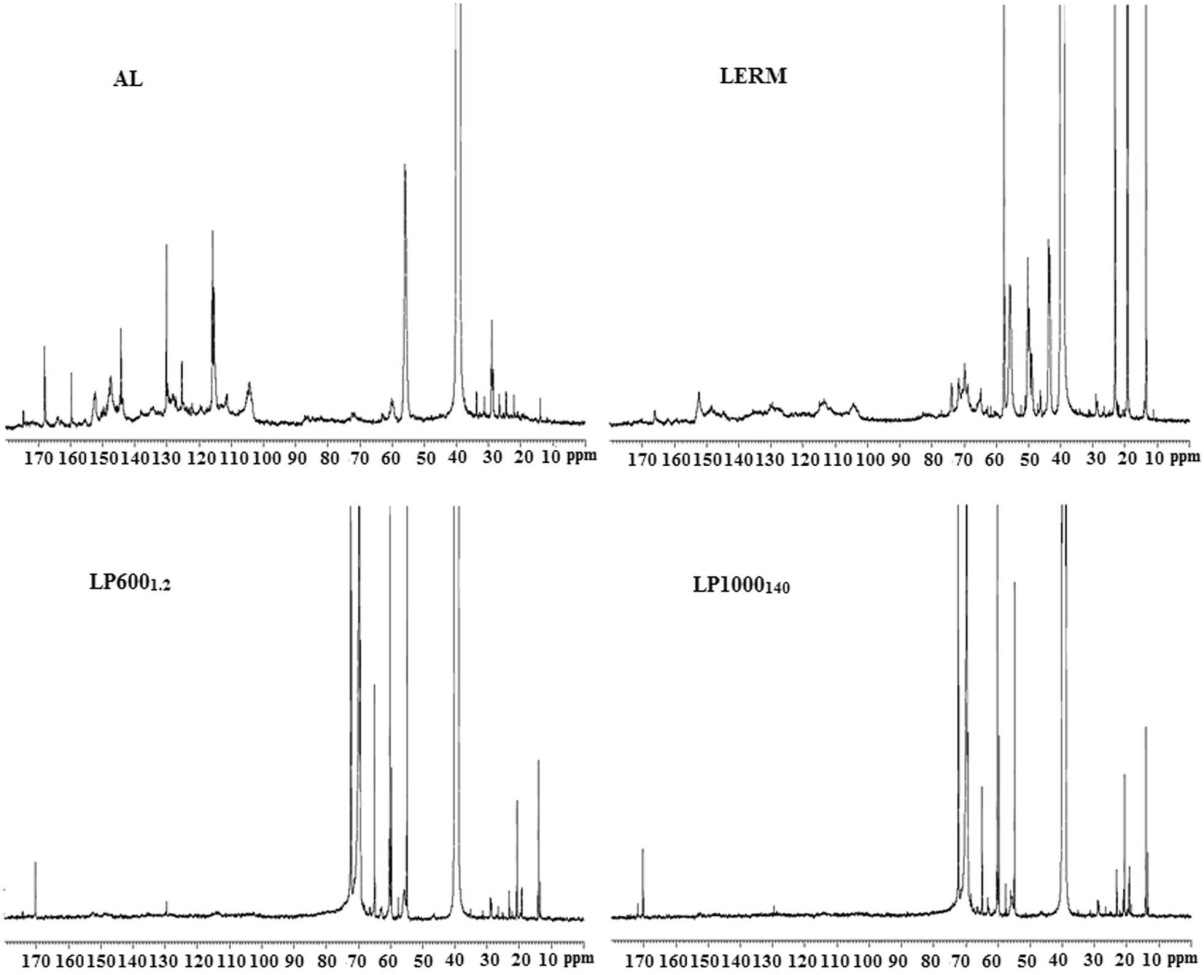
^13^C-NMR spectra of AL, LERM, LP600_1.2_, and LP1000_140_

Figure 3 represents the ^1^H-NMR spectra of AL, LERM LP600_1.2_, and LP1000_130_. The major distinction between AL and LERM was the epoxy groups. Because of the epoxidation of AL, the three new strong signals at 2.68, 3.16, and 3.37 ppm in the spectrum of LERM clearly represented the existence of epoxy groups in LERM, which is consistent with the results of FT-IR analysis. The information on the structural change and the synthesis reactions of LPEGs could be obtained from the comparison of LPEGs with AL and LERM. The methylene protons of PEGs (–CH_2_–O–CH_2_–, –CH_2_–OH) were observed at the new strong peaks at 3.41–3.51 ppm, indicating the successful introduction of PEGs into LERM. Additionally, a propyl structure (originated from epichlorohydrin) connected to the phenol oxygen of lignin was derived from the ring-opening reaction of epoxy groups of LERM. Two new peaks at 3.99 and 4.09 ppm in the LPEGs’ spectra are attributed to the protons of methylene connecting to phenol oxygen and the middle methylene of the propyl structure, respectively. The signals at 3.71–3.80 ppm corresponding to the protons of methoxy groups were observed in all the spectra, which revealed the stability of methoxyl groups connecting to the benzene ring in lignin. As can be seen, the signals at 6.31–7.51 ppm corresponding to the aromatic ring protons were weaker in LERM as compared to AL, resulting from the epoxidation of lignin. To further introduce PEGs into LERM and the oxidation depolymerization of lignin during the synthesis of LPEGs, the signals of aromatic ring protons displayed the weakest in the spectra of LPEGs. The difficulty of analyzing the lignin samples by ^1^H-NMR was mainly due to some overlapping signals. For example, the chemical shift at 2.49 ppm attributed to the protons of methyl (connecting to carboxyl groups) appeared in the spectra of all lignin samples, which cannot confirmed the oxidation depolymerization of products by the catalysis of KPS. Therefore, ^13^C-NMR was also used for further investigation. Figure 4 shows the ^13^C-NMR spectra of AL, LERM, LP600_1.2_, and LP1000_130_. The clear peaks at 170.3–170.7 ppm derived from carboxyl groups in the LPEGs revealed the oxidation depolymerization of lignin by the catalysis of KPS during the synthesis of LPEGs. The signals between 160.0 and 104.3 ppm are attributed to the aromatic structures of lignin. These signals showed significantly weaker correlation with the intensity in the LPEGs as compared to AL and LERM, which mainly resulted from the introduction of PEGs into lignin, which is consistent with the results of ^1^H-NMR analysis. Two types of hydroxyl groups (phenolic hydroxyl and alcoholic hydroxyl groups) in the raw lignin reacted with epichlorohydrin. After epoxidation of lignin, the carbon atoms in the epoxy groups, which formed ether linkages with these two types of hydroxyl groups in the lignin, showed different chemical shifts. The chemical shifts at 73.8 and 71.7 ppm in LERM contributed to the carbons of epoxy groups linking to oxygen atoms of phenolic and alcoholic hydroxyl groups, respectively. The other two carbon atoms in epoxy group were present in the peaks at 43.3–43.7 and 49.1–50.2 ppm, respectively. Furthermore, all the spectra of the lignin samples showed vibration absorption of methoxyl group carbon of lignin at 55.7–56.0 ppm. The strong signals at 71.7, 69.5–69.7, and 59.7–60.2 ppm are assigned to the methylene carbon (–CH_2_–O–CH_2_–, –CH_2_–OH) of PEGs in the LPEGs, suggesting the successful introduction of PEGs into lignin.

### Emulsifiability and surface activity of LPEGs

**Fig. 5:**
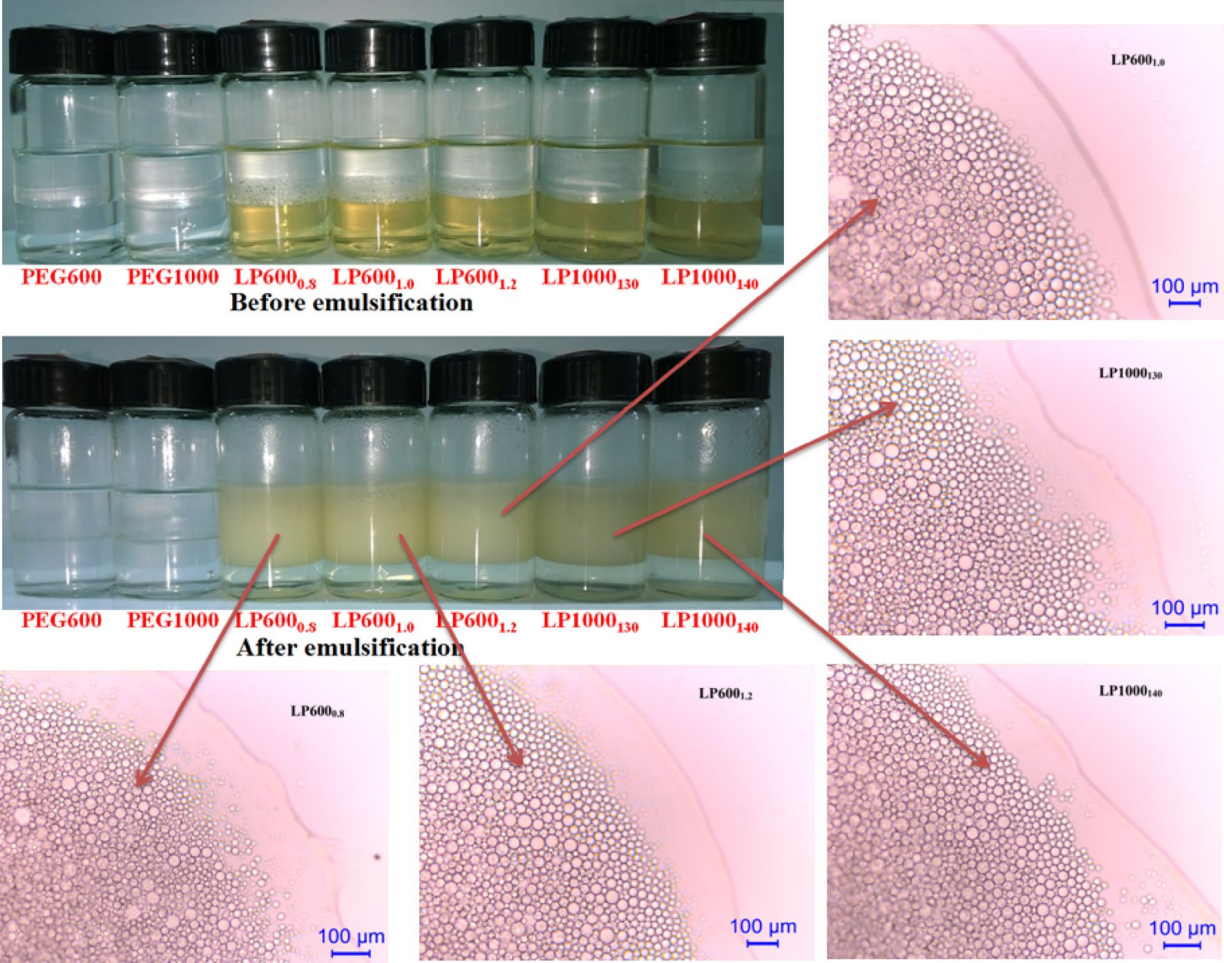
Images of the emulsification test of PEGs and LPEG samples before and after emulsifying treatment, and the emulsion droplet particle morphology under the observation of optical microscope. The *scale bar* at the *lower right corner* in each image represents 100 μm

To determine emulsion formation, all LPEGs were dissolved in deionized water at the concentration of 1 mg/mL, and then emulsions were formed by adding equal volume of *n*-hexanes followed by mixing with a vortex finder. The images of the emulsification test of PEGs and LPEG samples before and after emulsifying treatment are shown in Fig. 5. As can be seen, although PEG600 and PEG1000 possess both hydrophilic and lipophilic properties, the demulsification of emulsion layer in a short time after emulsifying treatment indicated that unstable emulsion layer of *n*-hexane and water was formed resulting from the poor emulsifiability of PEG600 and PEG1000. As compared to PEG600 and PEG1000, clear emulsion layers were observed by mixing LPEG solution and *n*-hexanes, and these emulsion layers were stable for at least 3 months in the sealing condition at room temperature. The volume fractions of emulsion layer were about 70 % in all LPEG samples (see Table 2), which indicated that LPEGs showed a significant emulsifiability as an emulsifier. Before emulsifying treatment, the water layer containing LPEGs presented canary yellow color and then changed into transparent and colorless after the treatment, which suggested that LPEGs had entered into the oil layer involved in the emulsification. For the granule shape of emulsion droplets in emulsion layer (see Fig. 5), there was no obvious difference in the physical size of the emulsion droplets, and droplet sizes of 10–50 μm were observed in the emulsion phases for all LPEG samples. Obviously, all the lignin samples with grafted PEGs had a similar emulsifying capacity. Thus, more detailed application performances of the emulsification should be tested in future. To compare the emulsifying effectiveness of the product LPEGs with the industrial surfactants, a comparison of emulsification test between Tween-80 and LP1000_140_ was also made (see Additional file 1: Figure S2). As shown, the similar volume fraction of emulsion and emulsion particle size of LP1000_140_ and Tween-80 suggested that the water-soluble lignin derivatives LPEGs have a good emulsifying capacity as an emulsifier.

**Table 2:** Volume fraction of emulsion, emulsion particle size, CMC, and surface tension at CMC of all LPEG samples

Sample^a^	Volume fraction of emulsion (%)	Emulsion particle size (μm)	CMC (%)	Surface tension of CMC (mN/m)
LP600_0.8_	71.4	10–50	1.05	44.0
LP600_1.0_	71.1	10–50	1.08	43.4
LP600_1.2_	70.3	10–50	1.00	49.3
LP1000_130_	70.8	10–50	1.03	43.3
LP1000_140_	69.4	10–50	1.03	43.5

^a^Corresponding to the samples in Table 1

**Fig. 6:**
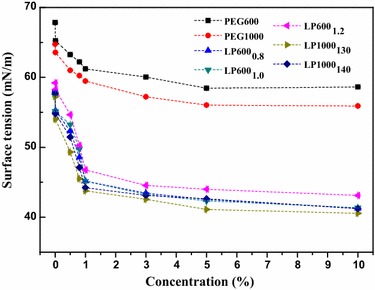
Isotherms of surface tension of water plotted against the concentration of LPEGs as compared to PEG600 and PEG1000. The *error bars* are standard deviations from experiments performed in triplicate

Generally, the surface activity of one surface-active substance was evaluated based on the ability to reduce surface tension of aqueous solution at the water–air interface. The effect on the capacity of LPEG samples reducing water surface tension was investigated at various concentrations. Figure 6 shows the surface tension–concentration isotherms for LPEGs as compared to PEG600 and PEG1000. Critical micelle concentration (CMC) values and the corresponding surface tension are summarized in Table 2. Similar CMC values of LP600_0.8_, LP600_1.0_, LP600_1.2_, LP1000_130_, and LP1000_140_ were found at 1.05, 1.08, 1.03, 1.03, and 1.03 %, respectively. Accordingly, the values of surface tension at CMC were found at 44.0, 43.4, 44.9, 43.3, and 43.5 mN/m, respectively. Commercial surfactant Tween-80 can reduce the air/water surface tension to about 44.0 mN/m at a CMC of 13.4 mg/L [33]. The synthetic lignin-based surfactants LPEGs in our study showed a close surface tension but at the relatively larger CMC, which could be due to the intrinsic disorganized structure of the lignin as well as the sodium lignosulphonate (a common lignin-based anionic surfactant) [34]. The surface activity of the products in this study was similar to that of amphiphilic lignin derivatives obtained from the modification of lignin with PEG diglycidyl ether in a previous report [35]. For LPEGs, the value of the surface tension was smaller than that of PEGs at the same concentration, which indicated that lignin with grafted PEGs significantly improved the surface activity of lignin. Moreover, LP600_1.2_ had a relatively higher surface tension as compared to LP600_0.8_ and LP600_1.0_ at the same concentration. This indicated a negative effect on the surface activity of product with the addition of a higher dosage of KPS. From the comparison between LP600_1.2_ and LP1000_130_, it was found that the introduction of a longer polyether chain into lignin in the same reaction condition was conducive to the surface activity of the products.

The results above indicated that the LPEGs had the application potential as an emulsifier, a detergent, and a dispersant. Further efforts should be made to investigate the application performances of LPEGs in the traditional fields, such as pesticide, cement, and dye. In addition, the water-soluble lignin derivatives with obvious emulsifying properties deriving from lignin epoxy monomers also provided a conception of the synthesis of water-soluble lignin epoxy resin.

### Effect of LPEGs on the enzymatic hydrolysis of lignocelluloses

**Fig. 7:**
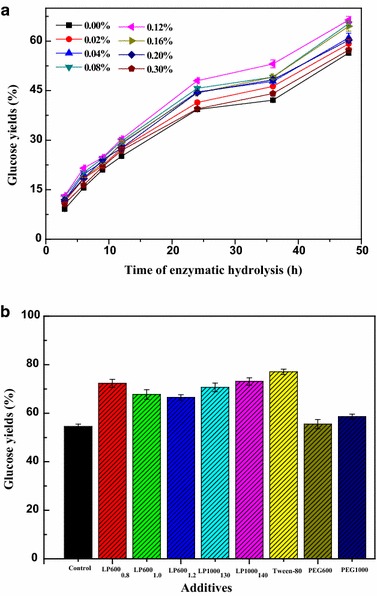
**a** Effect of enzymatic hydrolysis time and additive concentrations of LP600_1.2_ on enzymatic hydrolysis. **b** Glucose yields of the enzymatic hydrolysis at 48 h with different additives at 0.12 % concentration; the concentration of Tween-80 was set at 0.2 %. The *error bars* are standard deviations from experiments performed in triplicate

Biological conversion of lignocellulose to ethanol through enzymatic hydrolysis attracts widespread attention due to the advantages of significant environmental and economic benefits. However, the high production cost limited the development of this process due to the low glucose yield resulting from poor accessibility of enzymes to cellulose and high cellulase cost. Many pretreatment and post-treatment methods have been applied to enhance the enzyme hydrolysis of lignocelluloses in our laboratory, such as steam explosion [36], hydrothermal treatment [37, 38], ionic liquid treatment [39], alkaline treatment [40], acid treatment [41], organic solvent treatments [42], and integration treatments of these methods [43–45]. However, study on modification of lignin for the preparation of surfactants to enhance enzymatic hydrolysis is not available. Furthermore, many other researchers reported that common surfactants, especially commercial non-ionic surfactants (Tween and PEGs), have a significant capacity for enhancing the cellulose hydrolysis thus decreasing the enzyme loading to the reduce cost of bioethanol production [46–48]. As the lignin-based non-ionic surfactants, the capacity of enhancing enzymatic hydrolysis of the water-soluble products obtained from the grafting reaction of LERM with PEGs was also investigated and the hardwood pulp was used as the substrate. LP600_1.2_ was applied to investigate the effect of enzymatic hydrolysis time and additive concentrations (see Fig. 7a). At the beginning, all the digestion systems showed slow rates of enzymatic hydrolysis, whereas an obvious ascension of enzyme hydrolysis rate was observed from 9 to 12 h. The degree of the improvement of glucose yield increased from 54.5 to 66.5 % with the increase of the concentration of LP600_1.2_ from 0 to 0.12 %, whereas it further decreased to 57.3 % with the rise of the concentration of LP600_1.2_ to 0.30 %. Figure 7b shows the comparison of glucose yields of the enzymatic hydrolysis at 48 h between different additives (PEG600, PEG1000, and LPEGs). All the products obtained from the modification of lignin with PEGs showed higher glucose yields than that of PEG600 and PEG1000. The comparison among LP600_0.8_, LP600_1.0_, and LP600_1.2_ showed that the highest glucose yield of 72.2 % was achieved with LP600_0.8_, whereas the lowest one of 66.5 % was obtained from LP600_1.2_, which indicated that the relatively poor capacity for enhancing enzymatic hydrolysis was observed with the product obtained from the excessive catalyst. The products obtained from the reaction of LERM with PEG1000 exhibited better performance in enzymatic hydrolysis than those derived from the reaction of LERM with PEG600 in the same reaction condition. As compared to the glucose yield of LP600_1.2_, a relatively higher glucose yield of 70.6 % was obtained from LP1000_130_, which suggested that LP1000_130_ not only had a greater surface activity as mentioned previously but also showed a better capacity for enhancing enzymatic hydrolysis. In addition, although a relatively higher reaction temperature caused a stronger depolymerization of LP1000_140_ than that of LP1000_130_, a highest increasing rate of 18.6 % of glucose yield was observed in LP1000_140_ from 54.5 to 73.1 %. The appropriate increase of the temperature of the reaction of LERM with PEG1000 was in favor of improving the performance of the products in enhancing enzymatic hydrolysis. The results above showed that the effect of LPEGs on the enzymatic hydrolysis was similar to that of amphiphilic lignin surfactants derived from the modification of lignin with PEG diglycidyl ether on enhancing the enzymatic hydrolysis of unbleached softwood pulp [30]. Furthermore, Xu et al. [24] reported that lignin-based polyoxyethylene ether deriving from PEG-chlorohydrin-grafted enzymatic hydrolysis lignin enhanced the glucose yield of corn stover from 16.7 to 70.1 %, while the increase in yield with PEG4600 alone was 52.3 %. In order to compare the effect of commercial surfactant on the enzymatic hydrolysis with LPEGs, Tween-80 was also applied to the enzymatic hydrolysis as an additive. 77.0 % of the glucose yield of Tween-80 suggested a greater ability of Tween-80 for enhancing enzymatic hydrolysis yield compared with the synthesized non-ionic lignin surfactants.

It is worth noting that the LP1000_130_ has a better surface activity than that of LP600_0.8_ and LP1000_140_. LP600_0.8_ and LP1000_140_ showed a greater capacity for enhancing enzymatic hydrolysis yield. This indicated that there was no direct correlation between the surface tension of LPEGs and enzymatic hydrolysis. Similar results of the effect of amphiphilic lignin derivatives on the enzymatic hydrolysis were reported by Uraki Y et al. [35]. In their work, the DAEO-based lignin derivative obtained from the reaction of dodecyloxy-polyethylene glycol glycidyl ether and lignin showed a higher surface activity than EPEG-based lignin derivative obtained from the reaction of ethoxy (2-hydroxy)propoxy polyethylene glycol glycidyl ether and lignin, while the latter had a larger positive effect on enzymatic hydrolysis. In the present work, it was found that the optimal concentration of LP600_1.2_ (0.12 %) was less than the CMC. Furthermore, when the additive concentration was conducted at CMC, an inhibition effect of LP600_1.2_ on the enzymatic hydrolysis occurred (data not shown). Xing et al. [49] investigated the effects of Gleditsia saponin (a natural non-ionic surfactant) on enzymatic hydrolysis yield of furfural residues. They found that the optimal dosage of saponin was 25-fold greater than its CMC. Furthermore, all LPEGs show a similar CMC, while different improvement of enzymatic hydrolysis yields was obtained. The results above indicated that the effect of LPEGs on the enhancement of enzymatic hydrolysis yields was dependent upon many factors. Various explanations of the mechanism of surfactant effect on enzymatic hydrolysis have been proposed. The dominating mechanism to the positive effect of surfactant on enzymatic hydrolysis can be summarized as follows: (1) non-productive and irreversible binding of enzymes to lignin in the lignocelluloses decreases because the hydrophobic sites of the lignin are occupied by the surfactant. Furthermore, the hydrophilic groups of the surfactant will in turn protrude into the aqueous solution and cause steric repulsion of enzyme from the lignin surface [50]. (2) Surfactants play a role as an enzyme stabilizer, which effectively prevented enzyme from denaturation during hydrolysis and increased accessibility of the substrate [51, 52]. Thus, except for the decrease of non-productive and irreversible binding of enzymes to lignin by the interaction of lignin and surfactants, the main mechanism of the positive effect of LPEGs on the enzymatic hydrolysis yields should be due to the increase of the cellulase activity and stability as well as the increased accessibility of the substrate during the hydrolysis process.

### Effect of LPEGs on ethanol fermentation of lignocelluloses

**Fig. 8:**
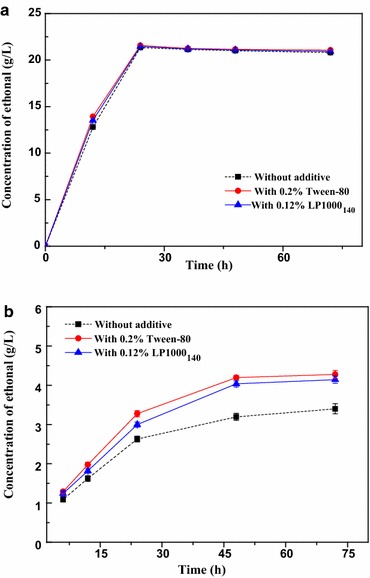
Influence of LP1000_140_ and Tween-80 addition on the ethanol production by **a** glucose fermentation and **b** SSF of lignocelluloses. The *error bars* are standard deviations from experiments performed in triplicate

The ultimate goal of enhancing enzymatic hydrolysis yield through the addition of LPEGs was the improvement of bioethanol yield through fermentation. It is important to test the effect of LPEGs on fermentation micro-organisms. So, an ethanol fermentation test was conducted to check whether the products affect fermentation micro-organisms or not. LP1000_140_ was chosen as the additive for the study and the system of fermentation without additive was used as the control. To test the effectiveness of LP1000_140_ as compared to the commercial surfactant, Tween-80 was also applied to the fermentation using 0.2 % concentration based on the previous report [53]. Prior to SSF, the effect of additives on glucose fermentation was investigated. Figure 8a shows the ethanol concentration produced by the ethanol fermentation of glucose (5 % of substrate) with and without surfactants. As can be seen, the ethanol concentration of glucose fermentation with surfactants was slightly higher than that of the reference run without surfactant for 12 h. When the fermentations run for 24 h, all the samples reached a close maximum ethanol concentration. This result indicated that lignin surfactant prepared in this study did not suppress the yeast fermentation as well as the commercial surfactant Tween-80. Figure 8b shows the ethanol concentration produced from the SSF of lignocelluloses with and without surfactants. In the reference run without surfactant, the final ethanol concentration in the SSF of lignocelluloses was 3.4 g/L corresponding to 42.9 % of theoretical ethanol yield. Interestingly, with the LP1000_140_ addition at 0.12 %, the ethanol concentration was increased to 4.2 g/L corresponding to 53.5 % of theoretical ethanol yield. The ethanol yield was increased by about 23.6 % with the addition of 0.12 % LP1000_140_. As compared to LP1000_140_, the commercial surfactant Tween-80 had a similar ability for improving fermentation which gave the relatively higher ethanol concentration at 4.4 g/L corresponding to 55.2 % of theoretical ethanol yield. The difference of ethanol yield of Tween-80 and LP1000_140_ can be explained by the difference in the ability to improve enzymatic saccharification. The results above indicated that LPEGs have no toxicity for fermentation micro-organisms and have similar positive effects on SSF as well as Tween-80. The water-soluble lignin surfactants were promising additives for enhancing the bioethanol fermentation yield. It is important for the high-value utilization of technical lignin in the wood chemistry field. However, more work on the development of process integration and optimization of process parameters is necessary to improve the ethanol concentration and yield.

## Conclusions

A new method was applied to prepare water-soluble lignin derivatives by epoxidation and etherification. About 70 % stable emulsion layer was formed with *n*-hexanes and droplet sizes of 10–50 μm in the emulsion layer were observed in the emulsion phases for all products, which indicated that the products showed a significant emulsifiability as an emulsifier. The lowest surface tension of 43.30 mN/m was achieved at 1.03 % concentration of LP1000_130_ solution, suggesting that water-soluble lignin derivatives with a good surface activity were obtained. A highest increasing rate of 18.6 % of glucose yield was observed in the product LP1000_140_ derived from the reaction of LERM with PEG1000. The results of fermentation experiments showed that there was no toxicity of the product for fermentation micro-organisms. The water-soluble lignin derivatives prepared in this study are a promising feedstock for detergents, emulsifier, and additives to enhance enzymatic hydrolysis efficiency and ethanol fermentation yield.

## Methods

### Materials

Alkaline lignin (AL) from corncob was provided by Shandong Longlive Bio-technology Co., Ltd, China. The content of aliphatic OH, phenolic OH, COOH, and total OH of the alkaline lignin were 2.26, 1.52, 2.24, and 8.03 mmol g^−1^ lignin, respectively [6]. Epichlorohydrin (ECH), tetrabutyl ammonium bromide (TBAB), potassium persulfate (KPS), polyethylene glycol (PEG600 and 1000), dichloromethane, petroleum ether, ethyl acetate, acetic acid, sodium hydroxide, and Tween-80 were purchased from Sinopharm Chemical Reagent Beijing Co., Ltd, China. Poplar pulp was obtained from Taian Paper Mill, Shandong, China. The main composition of the pulp was 70 % of cellulose, 25 % of hemicelluloses, 1.5 % of lignin, and 0.5 % of ash, determined according to the National Renewable Energy Laboratory method [54]. The pulp was smashed into uniform floccules before use.

### Enzyme and microorganism

Cellulase from *T. reesei* ATCC 26921 (Cellulast 1.5 L, C2730) containing ≥700 U/g exo- and endo-glucanases, and cellobiase from *Aspergillus niger* (Novozyme 188, C-6105) containing ≥250 U/g *β*-glucosidase were purchased from Sigma-Aldrich. The enzyme activity of Cellulast 1.5 L and Novozyme 188 was described as filter paper activity units (FPU) and cellobiase units (IU), respectively. Cellulast 1.5 L had an activity of 115 filter paper units (FPU)/mL measured using the IUPAC protocol [55], and Novozym188 had an activity of 560 IU/mL *β*-glucosidase activity determined according to the method of Berghem and Pettterson [56]. The microorganism used for fermentation was Saccharomyces cerevisiae in the form of dry yeast (thermal resistant) (Angel Yeast Company Ltd, Yichang, China). Dry yeast was activated in 2 % glucose solution at 40 °C for 30 min, then at 34 °C for 2 h.

### Preparation of lignin-based epoxy resin monomer (LERM)

A total of 100 g ECH was put into a three-neck round-bottom flask, and then 10 g AL and 5 g TBAB were added into the ECH at room temperature under mechanical stirring. The obtained mixture was kept at 90 °C for 5 h. Next, 65 mL of 48 % sodium hydroxide aqueous solution was added dropwise into the aforementioned mixture at room temperature under vigorous stirring, and the reaction was sequentially run for 5 h. After the reaction, the resulting solution was neutralized with 50 % acetic acid solution, and the organic layer was washed 4 times with 1 L of distilled water, separated, and dried with anhydrous sodium sulfate. The organic layer was then concentrated to 30 mL at 50 °C under reduced pressure and diluted with 70 mL dichloromethane. Furthermore, the diluent was precipitated into petroleum ether, filtered, and dried in ventilation places at room temperature to obtain the lignin-based epoxy resin monomer.

### Preparation of water-soluble lignin derivatives (LPEGs)

After 2 g LERM was completely dissolved into 40 g PGEs (PGE600 and PEG1000) in a 100-mL three-neck round-bottom flask at 60 °C by mechanical mixing, 0.8–1.2 g of potassium peroxydisulfate was added into the mixture, and then the reaction vessel was heated to 130–140 °C by oil bath for 5 h under atmospheric pressure. After the reaction, the resulting mixture was centrifuged and filtrated to remove the catalyst, yielding a transparent liquid. Finally, the liquid was precipitated into ethyl acetate/petroleum ether (v/v, 3/1) solvent and air dried to obtain LPEGs. All the samples were dissolved in a certain volume of dichloromethane and precipitated into ethyl acetate/petroleum ether solvent until the remaining PEGs were removed completely. Thin-layer chromatography (TLC) method with silica gel G plate was used to monitor the complete removal of remaining PEGs in the products. The plate was developed in a developing solvent chloroform/methanol (v/v, 2/1) following development in iodine vapor. PEG600 and PEG1000 were used as the blank controls.

### Sample characterization

Fourier transform infrared spectroscopy (FT-IR) spectra were collected using a Thermo Scientific Nicolet iN10 FT-IR Microscope (Thermo Nicolet Corporation, Madison, WI, USA). Each spectrum was recorded in 32 scans ranging from 4000 to 650 cm^−1^ at 4 cm^−1^ resolution. Nuclear magnetic resonance spectroscopy (NMR) spectra were obtained on a Bruker AVIII 400 MHz spectrometer at 25 °C. For NMR spectra, 100 mg sample was dissolved in 1 mL of DMSO-*d*_6_. The ^1^H-NMR spectrum was recorded at 100 MHz after 526 scans. A 30° pulse flipping angle, a 3.98 s acquisition time, and 1 s relaxation delay time were used. The ^13^C-NMR experiment was conducted at 400 MHz with 30,000 scans. A 30° pulse flipping angle, 9.2 μs pulse width, 1.36 s acquisition time, and 2 s relaxation delay time were used.

Gel permeation chromatography (GPC) analysis was conducted on a Waters 2695 separation module with a Waters 2998 photodiode array, a Waters 2414 refractive index detector, and two Waters Styragel 5 μm, HR 4E 7.8 × 300 mm column in series. The mobile phase used was HPLC-grade THF, and the flow rate was 1.2 mL/min. Calibration curve was generated by narrow disperse polystyrene standard from 156,000 to 580 Da in THF.

The PEG content of the LPEGs was measured through the UV spectrophotometric method according to the procedure in a previous report with minor modification [29]. Before determination, the samples were dissolved in alkaline deionized water (pH = 11) with a certain concentration. The AL calibration curve was obtained through the equation *Y* = 0.0327X−0.0004, *R*^2^ = 0.999 (*Y* = absorbance, *X* = concentration, mg/L), which was calculated from the absorbance at 280 nm of a series of AL solutions from 5 to 30 mg/L. 10 mg/L of LPEG solution was prepared to measure the lignin content using the calibration curve.

### Properties of LPEGs as surfactant and emulsifier

An automatic surface tensiometer (QBZY-2, Shanghai Fangrui Instrument Co., Ltd) was used to measure the surface tension of the surfactant solution at different concentrations at room temperature. Deionized water (surface tension at 72.12 mN/m) was employed for reference. PEG600 and PEG1000 were used as the control groups. Emulsification test was conducted by mixing 6 mL of PEG and LPEG solution (1 mg/mL) with equal volume of *n*-hexane at 3000 rpm for 3 min using a vortex shaker, and 24 h was allowed for the equilibration of the mixture to occur. The heights of water, oil, and emulsion layer were measured after equilibration. The physical size of the emulsion droplet particles in emulsion layer was observed with a Leica DM2500 optical microscope.

### Enzymatic hydrolysis and ethanol fermentation

10 mL of 2 % (w/v) bleached poplar pulp was dispersed into a 50 mM sodium acetate–acetic acid buffer (pH 4.8) with different dosages of additives (PEG600, PEG1000, and LPEGs). Then the mixtures were added into 25-mL Erlenmeyer flasks, and the flasks were kept at 50 °C in a reciprocating shaker at 150 rpm. Cellulase loading of 10 FPU/g substrate was used in the enzymatic hydrolysis of all samples. *β*-glucosidase was applied to limit the end-product inhibition with an activity ratio of cellobiase units to filter paper units of 1:2. And 0.15 mL of enzymatic hydrolysate was periodically withdrawn from the reaction mixture. The glucose yield was analyzed by high-performance anion exchange chromatography (HPAEC) according to the previous report [43]. LP600_1.2_ was applied to investigate the effects of enzymatic hydrolysis time and additive concentrations on glucose yields. The comparison of glucose yields of the enzymatic hydrolysis among all the additives (PEG600, PEG1000, and LPEGs) was made using the same enzymatic hydrolysis time and additive concentration.

Simultaneous saccharification and fermentation (SSF) test was performed at the conditions based on the previous reports [57] with minor modifications. 0.4 g substrate was added to 20 mL of 50 mM sodium acetate buffer (pH = 4.8) with or without additive in 50-mL Erlenmeyer flasks, followed by supplementation of 10 g/L yeast extract and 20 g/L peptone. Commercial cellulase (Cellulacst 1.5 L, 10 FPU/g substrate), *β*-glucosidase (Novozyme 188, 20 IU/g substrate), and Saccharomyces cerevisiae (3.0 g/L) were added, and then the system was incubated at 40 °C in a shaker at 120 rpm. Aliquots of 0.5 mL were withdrawn and centrifuged at 10,000 rpm for 5 min, and the supernatants were subjected to ethanol analysis. The concentrations of ethanol were determined by high-performance liquid chromatography (HPLC) system (Agilent 1200 series, Agilent Technologies, USA). The bioethanol yield (%) on theoretical maximum ethanol production was calculated according to the previous report [30].

## Additional file

10.1186/s13068-016-0499-9 **Figure S1**. The solubility of sodium lignosulphonate (LS) and alkaline lignin (AL) in epichlorohydrin before (a) and after (b) adding EDTA. **Figure S2**. The comparison of emulsification tests between LP1000_140_ and commercial surfactant Tween-80. The scale bar at lower right corner in each image represents 100 μm.

## Abbreviations

AL: alkaline lignin
HCl: hydrogen chloride
NaOH: sodium hydroxide
DMSO: dimethylsulfoxide
DMF: dimethylformamide
ECH: epichlorohydrin
TBAB: tetrabutyl ammonium bromide
KPS: potassium persulfate
PEGs: polyethylene glycols
PEG600: polyethylene glycol 600
PEG1000: polyethylene glycol 1000
LPEGs: water-soluble lignin derivatives
LERM: lignin-based epoxy resin monomer
FT-IR: Fourier transform infrared spectroscopy
NMR: nuclear magnetic resonance spectroscopy
CMC: critical micelle concentration
TLC: thin-layer chromatography
GPC: gel permeation chromatography
HPLC: high-performance liquid chromatography
HPAEC: high-performance anion exchange chromatography
UV: ultraviolet spectrum
Mw: mass-average molecular weight
Mn: number-average molecular weight
SSF: simultaneous saccharification and fermentation
