# Supplementary material for: Epoxidation and etherification of alkaline lignin to prepare water-soluble derivatives and its performance in improvement of enzymatic hydrolysis efficiency
Source: Biotechnol Biofuels. 2016 Apr 14;9:87. doi: 10.1186/s13068-016-0499-9 (PMC4832561; doi:10.1186/s13068-016-0499-9)
Supplement: Supplementary file 1 — 10.1186/s13068-016-0499-9 Figure S1. The solubility of sodium lignosulphonate (LS) and alkaline lignin (AL) in epichlorohydrin before (a) and after (b) adding EDTA. Figure S2. The comparison of emulsification tests between LP1000140 and commercial surfactant Tween-80. The scale bar at lower right corner in each image represents 100 μm. [file 13068_2016_499_MOESM1_ESM.docx]

**Additional file 1:**

**Epoxidation and etherification of alkaline lignin to prepare water-soluble derivatives and its performance in** **improvement of enzymatic hydrolysis efficiency**

Changzhou Chen ^1^

Email: [ccz1988521@126.com](mailto:ccz1988521@126.com)

Mingqiang Zhu ^1,2^

Email: [zmqsx@nwsuaf.edu.cn](mailto:zmqsx@nwsuaf.edu.cn)

Mingfei Li ^1^

Email: [limingfei@bjfu.edu.cn](mailto:limingfei@bjfu.edu.cn)

Yongming Fan ^1^

Email: [fanym@bjfu.edu.cn](mailto:fanym@bjfu.edu.cn)

Run-Cang Sun ^1*^

Email: rcsun3@bjfu.edu.cn

^1^ Beijing Key Laboratory of Lignocellulosic Chemistry, Beijing Forestry

University, Beijing 100083, China;

^2^ College of Forestry, Northwest A&F University, Yangling 712100, China

* Correspondence: Beijing Key Laboratory of Lignocellulosic Chemistry, Beijing Forestry University, Beijing, 100083, China

**Results and discussion**


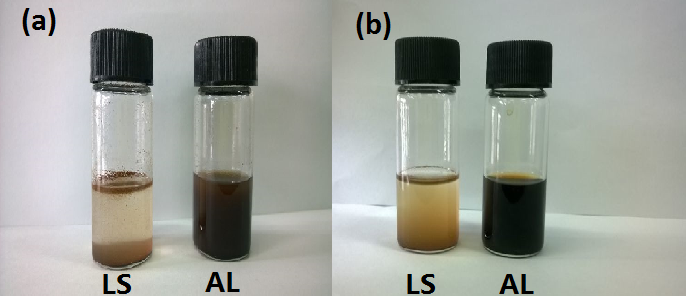


**Figure S1.** The solubility of sodium lignosulphonate (LS) and alkaline lignin (AL) in epichlorohydrin before (a) and after (b) adding EDTA.

As seen, lignosulfonates was difficultly dissolved in epichlorohydrin before and after adding EDTA, which resulted in the inadequate chemical reaction between lignosulfonates and epichlorohydrin. As compared to LS, alkaline lignin was easily dispersed into epichlorohydrin than LS before adding EDTA and can be completely dissolved in epichlorohydrin after adding EDTA in the reaction system. Hence, to ensure the sufficient reaction of lignin with epichlorohydrin, the alkaline lignin was chose as the raw material for chemical modification in the present work.


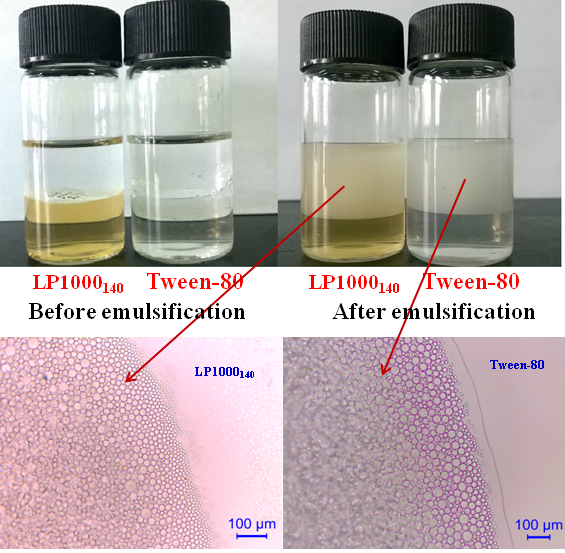


**Figure S2****.** The comparison of emulsification tests between LP1000_140_ and commercial surfactant Tween-80. The scale bar at lower right corner in each image represents 100 μm.

Emulsification tests between LP1000_140_ and Tween-80 was conducted by 6 mL of LP1000_140_ and Tween-80 solution (1 mg/mL) mixing equal volume of n-hexane at 3000 rpm for 3 min using a vortex shaker, and 24 h was allowed for the equilibrium of the mixture. As see, the similar volume fraction of emulsion and emulsion particle size of LP1000_140_ and Tween-80 indicated that the water-soluble lignin derivates LPEGs has a good emulsifying capacity as emulsifier.
